# *Plasmodium falciparum* parasites with histidine-rich protein 2 (*pfhrp2*) and *pfhrp3* gene deletions in two endemic regions of Kenya

**DOI:** 10.1038/s41598-017-15031-2

**Published:** 2017-11-07

**Authors:** Khalid B. Beshir, Nuno Sepúlveda, Jameel Bharmal, Ailie Robinson, Julian Mwanguzi, Annette Obukosia Busula, Jetske Gudrun de Boer, Colin Sutherland, Jane Cunningham, Heidi Hopkins

**Affiliations:** 1 London School of Hygiene and Tropical Medicine, London, UK; 2 Centre for Statistics and Applications of University of Lisbon, Lisbon, Portugal; 3 International Centre of Insect Physiology and Ecology, Nairobi, Kenya; 4 Laboratory of Entomology, Wageningen University, Droevendaalsesteeg 1, 6708 PB, Wageningen, The Netherlands; 5 Global Malaria Programme, World Health Organization (WHO-GMP), Geneva, Switzerland; 6 Present Address: Kaimosi Friends University College, Kaimosi, Kenya; 7 Present Address: Netherlands Institute of Ecology, Droevendaalsesteeg 10, 6708 PB, Wageningen, The Netherlands

## Abstract

Deletions of the *Plasmodium falciparum hrp2* and *hrp3* genes can affect the performance of HRP2-based malaria rapid diagnostic tests (RDTs). Such deletions have been reported from South America, India and Eritrea. Whether these parasites are widespread in East Africa is unknown. A total of 274 samples from asymptomatic children in Mbita, western Kenya, and 61 genomic  data from Kilifi, eastern Kenya, were available for analysis. PCR-confirmed samples were investigated for the presence of *pfhrp2* and *pfhrp3* genes. In samples with evidence of deletion, parasite presence was confirmed by amplifying three independent genes. We failed to amplify *pfhrp2* from 25 of 131 (19.1%) PCR-confirmed samples. Of these, only 8 (10%) samples were microscopic positive and were classified as *pfhrp2*-deleted. Eight microscopically-confirmed *pfhrp2*-deleted samples with intact *pfhrp3* locus were positive by HRP2-based RDT. In addition, one PCR-confirmed infection showed a deletion at the *pfhrp3* locus. One genomic sample lacked *pfhrp2* and one lacked *pfhrp3*. No sample harbored parasites lacking both genes. Parasites lacking *pfhrp2* are present in Kenya, but may be detectable by HRP-based RDT at higher parasitaemia, possibly due to the presence of intact *pfhrp3*. These findings warrant further systematic study to establish prevalence and diagnostic significance.

## Introduction

Antigen-detecting RDTs play a key role in malaria control successes in many parts of the world, and the global availability and scale of use of RDTs has increased dramatically over the last 10 years^1^. Most tests currently in use detect *Plasmodium falciparum* histidine-rich protein 2 (HRP2) and/or plasmodium lactate dehydrogenase (pLDH) antigens; data indicate that at least some RDT products also detect HRP3 due to a shared antigenic epitope^2,3^. Compared with RDTs that detect pLDH, in general HRP2-detecting RDTs are more sensitive and are less susceptible to degradation from heat and humidity during transport and storage^4^. In sub-Saharan Africa, which bears 90% of the global malaria burden, RDTs accounted for 74% of diagnostic testing among suspected cases in 2015, and HRP2-based tests are the most widely used^5^.

Performance of HRP2-based RDTs can be affected by factors including antigenic variability of the target protein, antigen persistence in the bloodstream following elimination of parasites, and parasite density below the RDT threshold of detection^3,6^. In one study in western Kenya, HRP2-based RDT gave false-negative results in 5–10% and 25–30% of samples compared to microscopy and PCR respectively^7^. Historically such results have been ascribed to varying thresholds of detection for the different tests.

More recently, concerns have been raised that such false-negative results may reflect presence of parasites with *pfhrp2* gene deletions or mutations. There has long been evidence of *P*. *falciparum* parasites lacking the *pfhrp2* gene in regions of South America^8^, and public health authorities have always cautioned against using HRP2-based RDTs in the Amazon River basin. In the past few years, reports have emerged of *pfhrp2* mutations or deletions in Africa and India^9–12^ leading to false-negative RDT results. More recently, in Eritrea, 80% of microscopically confirmed positive samples were negative by HRP2-based RDT, a finding attributed to deletion of the *pfhrp2* gene^13,14^.

The existence of parasites lacking *pfhrp2* would affect RDT accuracy in a broader range of malaria-endemic regions and would have significant implications for RDT implementation, clinical case management, and malaria control efforts^15^. Currently, there is little evidence to document the true extent of such mutations in Africa. Guidelines have recently been issued for study design and molecular assays to assess gene mutations^16^.

To extend current knowledge on the relationship between RDT failure and deletions of *pfhrp2* in African populations, we investigated samples from a cross-sectional survey in Mbita (western Kenya) and from published *P*. *falciparum* genome sequence data from Kilifi (eastern Kenya).

## Results

### Detection of *P*. *falciparum* malaria

**Figure 1:**
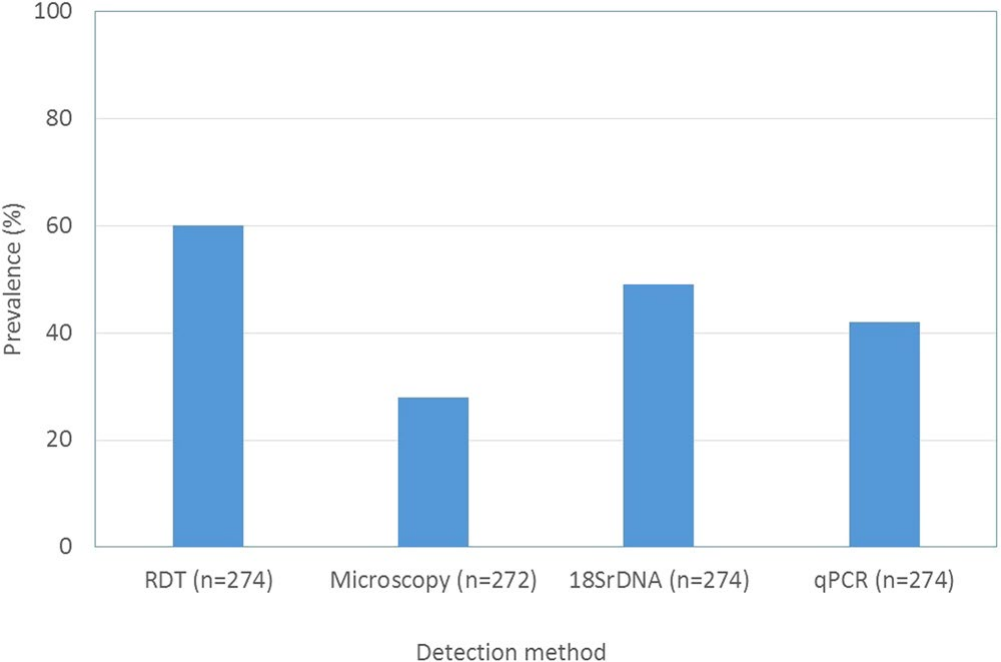
Proportion of different detection methods positive for malaria. Histidine-rich protein 2 (HRP2)-based rapid diagnostic tests (RDTs) generated more positive samples compared to other methods. PCR methods produced 13–20% more positive results compared to microscopy.

Of the 274 patient samples, 165 (60%) were positive by RDT. Of these, 114 (70%) were positive by HRP2 band only; 51 (31%) were positive by both HRP2 and pLDH bands. Microscopy detected parasites in 76/272 (28%) (Microscopy data were missing for two samples and of these, two (2.8%) were RDT negative. Of the 196 microscopic negative samples, 93 (52%) were RDT positives). The minimum parasite density reported by microscopy was 80 parasites/µL, suggesting that this was the threshold for detection by the field microscopist. Using *18SrDNA* PCR and qPCR assays, 131 (47.8%) and 114 (41.6%), respectively, were positive for *P*. *falciparum* (Fig. 1).

### PCR detection of *pfhrp2* and *pfhrp3*

**Figure 2:**
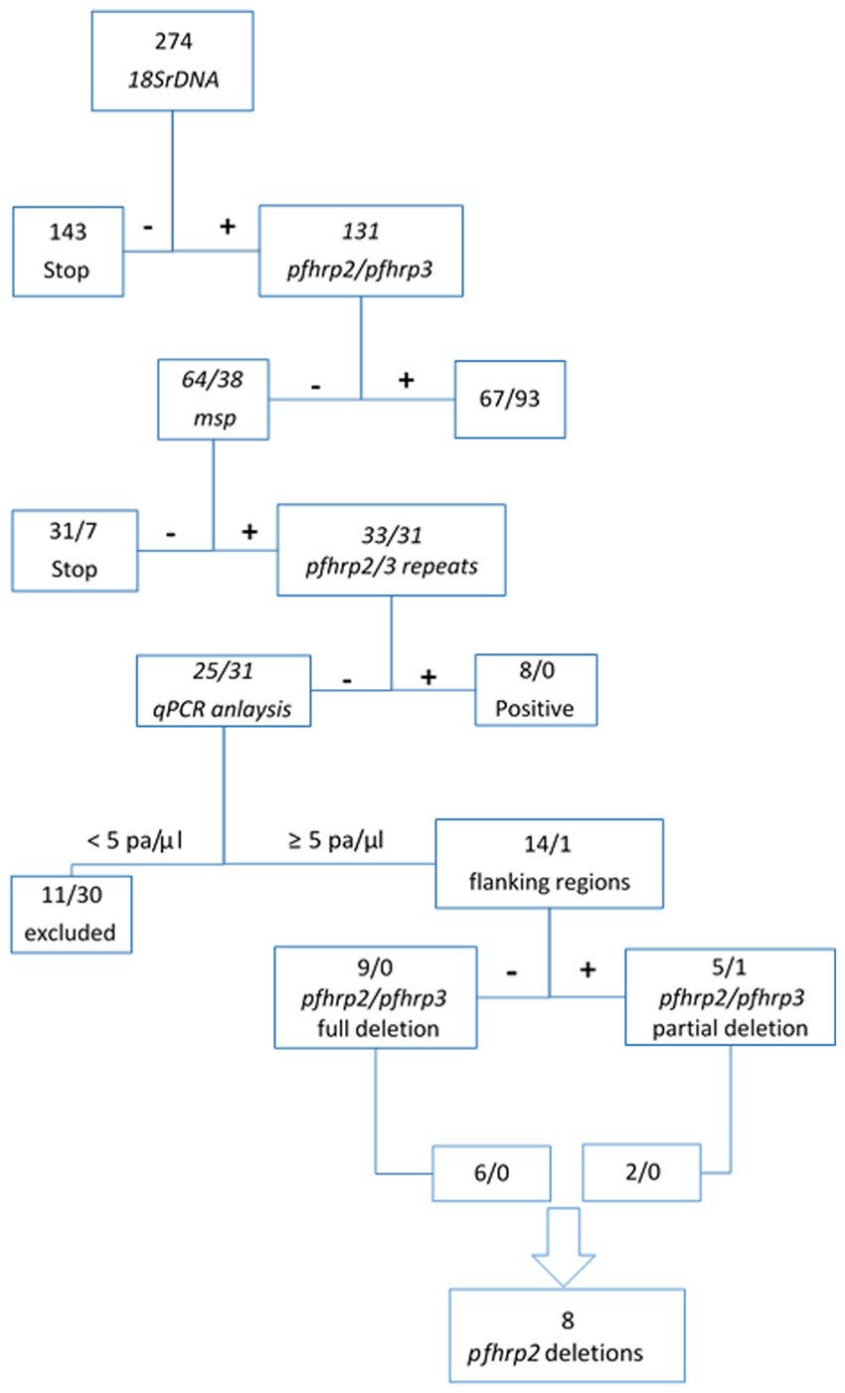
Flow diagram showing the strategy for determining deletion of *pfhrp2* and *pfhrp3* genes. Total of 274 Plasmodium falciparum samples were amplified using 18SrDNA PCR. All the positives (131 samples) were then subjected to *pfhrp2* and *pfhrp3* PCR amplifications. *Pfhrp2* and *pfhrp3* negative samples (64 and 38 respectively) were then further subjected to PCR amplification using *msp1* and *msp2* primers to confirm the presence of parasites. *Pfhrp2* and *pfhrp3* PCR amplification were repeated if the samples were *msp1*/*msp2* and 18SrDNA positive but *pfhrp2*/*pfhrp3* negative (33 and 31 respectively). A qPCR analysis was then performed on those samples negative by *pfhrp2* and *pfhrp3* but positive by other PCR methods (25 and 31 respectively). Samples with parasitaemia less than 5 parasites per microliter were excluded (11 and 30 respectively). The upstream and downstream flanking regions of *pfhrp2* and *pfhrp3* for the remaining samples were then amplified using specific primers (14 and 1 respectively). To reduce the risk of false *pfhrp2*-negative due to low parasite density, only microscopy positive samples were called *pfhrp2* and *pfhrp3* deletions (8 samples for *pfhrp2* only).

In this report, the term *pfhrp2*- or *pfhrp3*-negative is used for isolates that were negative by PCR for *pfhrp2* and/or *pfhrp3* in duplicate. The term *pfhrp2*- *or pfhrp3*-deleted is used for isolates that met the following four criteria: microscopy positive, three independent control genes amplified (*18SrDNA*, *msp1* and *msp2*), *pfhrp2*- or *pfhrp3*-negative in duplicate, and parasite density ≥5 p/μL. *Pfhrp2* genotyping of samples positive by *18SrDNA* produced 51% (67/131) positive and 10.6% (14/131) negative results. The remaining 38.2% samples (50/131) either had parasitaemia ≤5 p/μL by qPCR or were negative by *msp1*/*msp2* PCR and were excluded from further *pfhrp2* analysis. Therefore, only 14 of the *pfhrp2*-negative samples were included in further gene deletion call (Fig. 2). No significant difference in multiplicity of infection was observed between *pfhrp2*-deleted samples (n = 8, mean = 4.77, range 3–7) and *pfhrp2*-positive samples (n = 25, mean = 4.8, range 3–7).

*Pfhrp3* genotyping of samples positive by *18SrDNA* PCR gave a positive result for 71% (96/131). A single sample that was positive by all other criteria, including for *pfhrp2*, was negative for *pfhrp3*. Conversely, all 14 of the *pfhrp2* negative samples were *pfhrp3* positive.

### Association between parasitaemia and *pfhrp2* PCR results

To test whether parasitaemia affected the *pfhrp2* and *pfhrp3* PCR outcome, we compared the mean parasitaemia for samples that were positive by *18SrDNA* and qPCR, excluding samples with parasite density ≤5 p/μL by qPCR. Mean qPCR parasitaemia for *pfhrp2* positive and negative samples, respectively, was 688 ± 923 (n = 73, mean + SD) and 611 ± 1444 (n = 18, mean + SD) p/μL, a statistically significant difference (Kruskall-Wallis test, P = 0.01; Table 1). However, if only microscopy-positive samples are included, the difference is not significant (n = 65, p = 0.48). The same analysis was not done for *pfhrp3* as the negative sample size was only one.

**Table 1:** Correlation between pfhrp2 PCR result and Plasmodium falciparum parasitaemia.

	18SrDNA + ve N = 131	18SrDNA + ve > 5 parasite/μl N = 91	18SrDNA/msp1/msp2 PCR + ve > 5 parasite/ul N = 15	Mean parasite per µL (range) by qPCR^1^	Mean parasite/µL by microscopy^1^	P-value^2^
*pfhrp2* + RDT −	1 (0.8%)	1 (1.1%)	1	167	0	0.01^2*a*^ 0.48^2*b*^
*pfhrp2* + RDT +	74 (56.4%)	72 (79.1%)	NA	689 (8.2–3923)	1317 (0–7840)	
*pfhrp2−* RDT+	34(26%)	10 (11%)	8	1088 (8.5–6097)	1540 (240–3560)	
*pfhrp2−* RDT−	22 (16.8%)	8(8.8%)	6	10 (1.2–28.1)	0	

^1^For samples that were positive by both *18SrRNA* and qPCR, with qPCR parasitaemia ≥5 parasites per µL.^2^ Kruskal-Wallis rank test for^2a^ *pfhrp2*+ and *pfhrp2*− of all samples, and^2*b*^ for samples with microscopically detected parasitaemia (i.e. excluding samples with submicroscopic parasitaemia). S18rDNA, 18 ribosomal RNA subunit gene; *pfhrp2* and *pfhrp3*, *Plasmodium falciparum* histidine-rich protein 2 and 3 respectively; RDT, rapid diagnostic test.

### Confirmation of *pfhrp2* and *pfhrp3* deletions

QPCR parasite density was used to further investigate presence or absence of *pfhrp2* and *pfhrp3* genes in microscopy-negative samples. Of the total 15 samples included in the *pfhrp2* and *pfhrp3* deletion call, one was *pfhrp2*-positive and *pfhrp3*-negative with a parasite density of 167 p/μL and negative RDT result. The remaining 14 samples were *pfhrp2*-negative and *pfhrp3*-positive (*pfhrp2*−/*pfhrp3*+); of these, 42.8% (6/14) were RDT-negative. All six of these RDT-negative samples were also negative by microscopy suggesting that the parasitaemia was sub-microscopic. Fifty-seven per cent (8/14) of the *pfhrp2*−/*pfhrp3*+ and RDT-positive (either HRP only, or both HRP and pLDH) samples were also positive by microscopy with a mean density of 1540 ± 1123 p/μL and mean relative parasite density by qPCR of 1358 ± 1680. Therefore, 9% (8/89) of samples with adequate parasitaemia as measured by both microscopy and qPCR were *pfhrp2*-deleted (Table 2). In addition, 6.7% (6/89) and 1.1% (1/89) of qPCR-confirmed samples were *pfhrp2* and *pfhrp3*-negative, respectively. All the *pfhrp2*−/*pfhrp3*+ samples were positive by three separate PCR assays, two of which targeted single-copy genes (*18sDNA*, *msp1* and *msp2*).

**Table 2:** Plasmodium falciparum samples with *pfhrp2* or *pfhrp3* deletion. Nine samples positive by three independent PCR assays, two of which target single-copy genes, were negative by PCR for *pfhrp2* or *pfhrp3*. PCR outcome of the corresponding flanking regions are also shown. *Positive by qPCR with parasitaemia of 167 parasites per µL. *Pfhrp2* and *pfhrp3*, *Plasmodium falciparum* histidine-rich protein 2 and 3 respectively; RDT, rapid diagnostic test; PF3D7_0831900 and PF3D7_0831700, upstream and downstream flanking regions of *pfhrp2*, PF3D7_1372100 and PF3D7_1372400, upstream and downstream flanking regions of *pfhrp3*.

Isolate	RDT	Parasitaemia	PF3D7_0831900	*pfhrp2*	PF3D7_0831700	PF3D7_1372100	*pfhrp3*	PF3D7_1372400
K165	+	3560	−	−	+	+	+	+
K027	+	240	−	−	−	+	+	+
K065	+	1720	−	−	−	+	+	+
K031	+	2400	−	−	−	+	+	+
K063	+	2120	−	−	−	+	+	+
K233	+	840	−	−	−	+	+	+
K263	+	480	−	−	−	+	+	+
K320	+	960	−	−	+	+	+	+
K182	−	0*	+	+	+	−	−	+

### Relationship between RDT and parasitaemia of *pfhrp2*−/*pfhrp3*+ parasites

**Figure 3:**
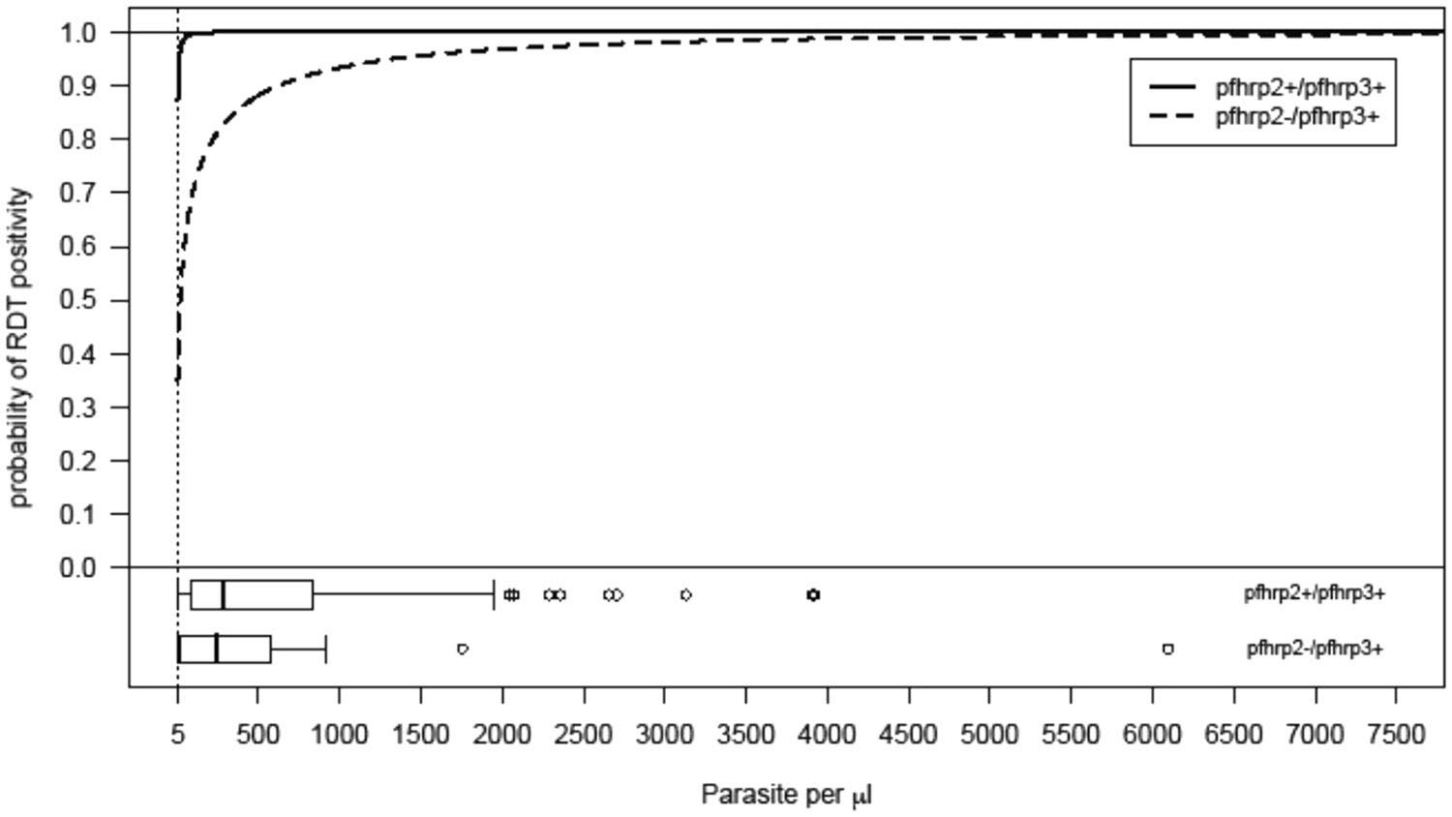
Complementary log-log regression model for the probability of RDT positivity as function of parasitaemia and *pfhrp2*/*pfhrp3* status in field samples from western Kenya with parasitaemia ≥5 parasite per μl (n = 91): The model shows the probability of RDT positivity in samples where both *pfhrp2* and *pfhrp3* genes are present (*pfhrp2*+/*pfhrp3*+, solid line) and in samples with *pfhrp2* deletion (*pfhrp2*−/*pfhrp3*+, dashed line) across a range of parasitaemia (*pfhrp2*−/*pfhrp3*- not included in the model as there were no such profile in the samples). The corresponding boxplots of parasitaemia as function of *pfhrp2*/*pfhrp3* status are also shown at the bottom of the plot.

To explore the possibility that differences in parasitaemia of the *pfhrp2*−/*pfhrp3*+ samples affect RDT results, samples were categorized by RDT result. Those *pfhrp2*−/*pfhrp3*+ samples that were RDT-positive had significantly higher parasite density (mean + SD, 1358 ± 1980 p/µL) compared to the RDT-negative samples (mean + SD, 17 ± 9, p = 0.0014). However, with available data it is not possible to determine whether these RDT were negative due to absence of *pfhrp2* or due to low HRP2 antigenemia resulting from low parasitaemia. To extend this analysis, generalized linear models including parasitaemia and *pfhrp2*/*pfhrp3* status as covariates were fit to the data from field samples with parasitaemia ≥5 μl (See Supplementary Table 1). The best model for the data was based on complementary log-log regression and it would appear to describe the data well (Hosmer-Lemeshow goodness-of-fit test, p = 0.90) and a good prediction power (area under the receiver operating characteristic curve = 0.96; 95% CI = (0.92–1)). According to this model for the data, the probability of RDT positivity for *pfhrp2*−/*pfhrp3*+ samples increased with parasitaemia and was close to the one for *pfhrp2*+/*pfhrp3*+ samples for parasitaemia levels >1000 μl (Fig. 3). Such proximity of the two probabilities together with the reduced number of *pfhrp2*−/*pfhrp3*+ samples implied that they were not statistically different as function of parasitaemia (effect of HRP3 = 1.09, SE = 0.73, p = 0.14; Supplementary Table 1).

### Analysis of *pfhrp2* and *pfhrp3* genes using genomic data from Kilifi

**Figure 4:**
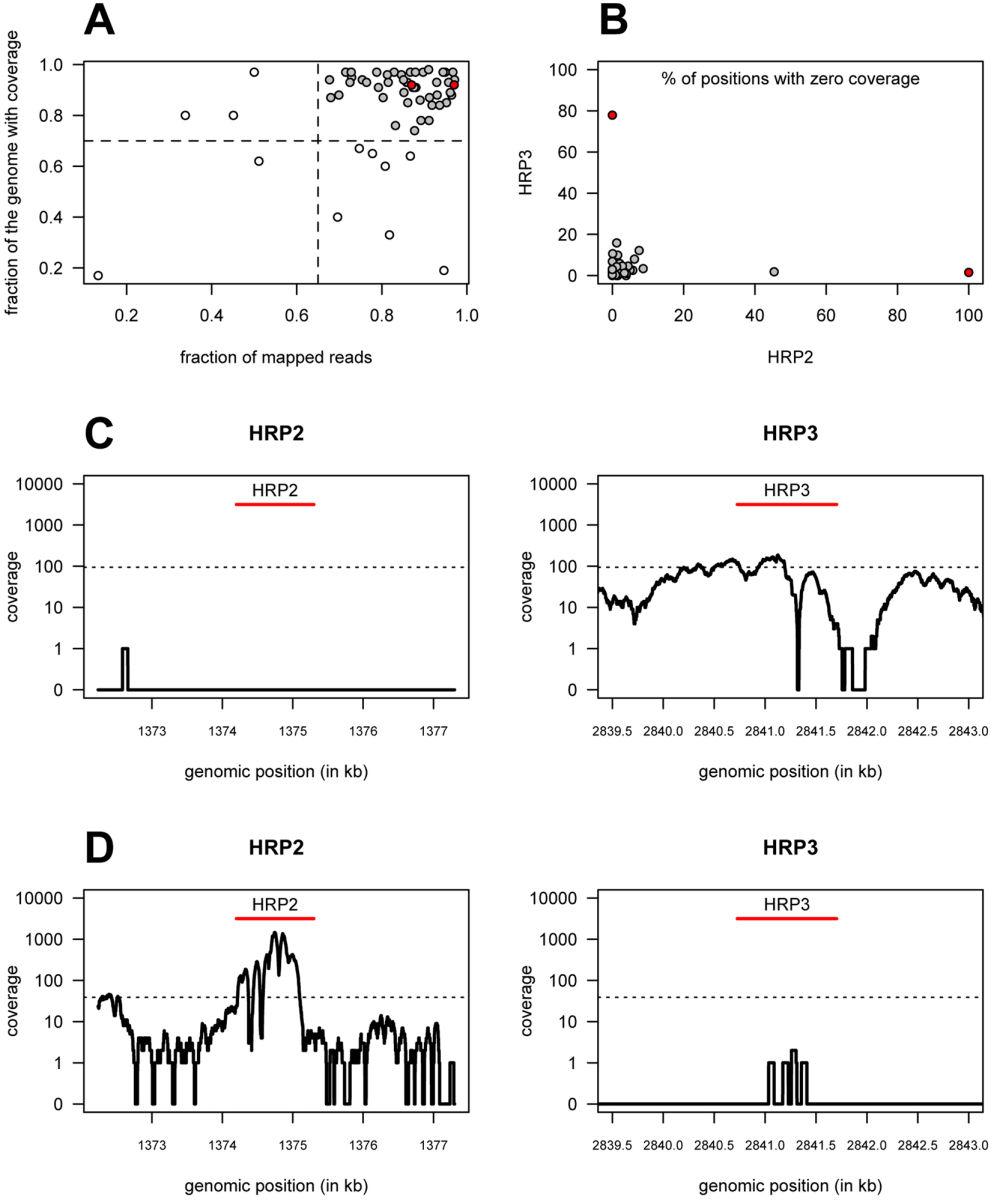
Coverage analysis of *pfhrp2* and *pfhrp3* genes using whole genome sequencing data from Kilifi, eastern Kenya. (**A**) Scatterplot of the percentage of genome with coverage (i.e., percentage of positions with at least one read mapped) versus percentage of reads that could be mapped on the 3D7 reference genome before quality checks (n = 61). Vertical and horizontal lines indicate the quality control cut-off used in the analysis while grey and white dots represent the genomes selected and not selected for the subsequent coverage analysis. (**B**) Scatterplot of the percentage of positions of *pfhrp2* and *pfhrp3* genes with no coverage after quality checks (n = 49). Red dots in A and B represent samples with possible evidence for a deletion in either gene. (**C** and **D**) Coverage profiles of the *pfhrp2* and *pfhrp3* genes and their respective 2 kb flanking regions to the either side for two genomes with possible evidence for a deletion in either gene, where dotted lines represent the average coverage of the whole genome in the respective samples.

According to the cutoff values for coverage in Fig. 4 (0.7 and 0.65 cutoff values for genome with coverage and mapped reads respectively), 12 of 61 genomes could not be mapped onto the 3D7 reference genome (Fig. 4A), and thus they were discarded from further analysis.

Among the 49 remaining genomes, only two showed a high percentage (>50%) of genomic positions with zero coverage in *pfhrp2* and/or *pfhrp3* genes (Fig. 4B). Data from one of these genomes revealed no coverage for the entire *pfhrp2* locus with the exception of coverage provided by a single read mapped onto the left flanking region, providing evidence for a possible deletion of this gene (Fig. 4C). In contrast, the *pfhrp3* locus shows some variations in coverage but mostly in a good agreement with the average coverage over the whole genome. This result suggests that the lack of coverage observed in the *pfhrp2* locus is unlikely to have resulted from problems in mapping these genes. Data from the other genome showed a possible deletion of the *pfhrp3* gene with only three reads mapped onto its extended locus (Fig. 4D). Given the good agreement between the coverage observed in the *pfhrp2* coding regions and the overall average coverage, the existence of three mapped reads onto the *pfhrp3* gene could have simply resulted from a mapping artifact due to sequence homology between the two genes. Finally, there is no evidence for the presence of genomes with possible deletions in both genes (Pearson-Clopper 95% confidence interval for the respective proportion: 0–7.3%).

## Discussion

This study provides evidence of *P*. *falciparum* lacking the *pfhrp2* gene in two areas of Kenya. Eight of 89 (9%) samples analyzed from western Kenya were *pfhrp2*-deleted; and genomic data from eastern Kenya identified a genome with no coverage for the *pfhrp2* gene or flanking regions. In addition, we have confirmed that parasite density is a factor both for PCR detection of *pfhrp2* and for determining RDT outcome in samples lacking *pfhrp2*, particularly in samples with submicroscopic parasitaemia. Of note, the microscopically-confirmed *pfhrp2*-deleted samples were positive by HRP2-based RDTs. This is surprising as previously reported parasites lacking *pfhrp2* were generally negative by HRP2-based RDTs regardless of *pfhrp3* status^2,8,10,17^ though some reported positive RDT results^8,18^.

The *pfhrp2*-negative samples included in this deletion call had microscopy-confirmed *P*. *falciparum* density of at least 250 p/μL. The integrity of the DNA in the samples and exclusion of PCR inhibitors was verified by successful amplification using three separate PCR assays, two of which targeted single-copy genes. One other possible cause for *pfhrp2*- or *pfhrp3*-negative is sequence variability of the region where the primers bind. However, the upstream and downstream segments of *pfhrp2* also gave negative results for at least six samples. Possible conclusions are that either these HRP2-based RDT results were false-positive, or the HRP signal was generated by protein expressed from *pfhrp3* or other genes; the latter explanation seems more plausible as explained below.

These results are in agreement with an *in vitro* study showing that the D10 parasite clone, which lacks *pfhrp2* ^19^, gave positive RDT results on two HRP2-based RDT at parasitaemia ≥1000 p/μL^8^. Six of the eight RDT-positive Kenyan samples with *pfhrp2* deletion had parasitaemia ≥890 p/μL (mean, 1358), which supports the *in vitro* finding. Sequence analysis of HRP2 and HRP3 revealed that they share similar alanine- and histidine-rich amino acid repeats and that four of the eight repeats found in HRP3 are identical to those of HRP2^2^. It is possible that antibodies raised against HRP2 cross-react with epitopes of HRP3 to generate an RDT signal, as previously reported^20^. However, the level of HRP3 contribution to the signal is unknown. It is also not clear whether the HRP3 expressed is enough to generate signal in the absence of HRP2 expression or if other proteins are involved, as Baker *et al*. observed no compensatory increase of *pfhrp3* transcription in the absence of *pfhrp2* ^21^. However, a study using yeast knockout collections reported that deletion of individual genes in a genome results in a genomic disturbance capable of driving the selection for mutations elsewhere in the genome^22^. The loss of *pfhrp2* may drive selection for mutations in *pfhrp3* or other similar genes particularly in the alanine- and histidine-rich amino acid repeats. This could be established by sequencing *pfhrp3* in parasites with and without *pfhrp2* and determining whether there is any sequence or repeat pattern difference. Overall, the genotypic data presented here together with the *in vitro* study elsewhere^8^ show that parasites with the *pfhrp2*−/*pfhrp3*+ genetic profile can produce a positive result on at least some brands of HRP2-based RDTs.

Genomic data from eastern Kenya samples also showed evidence for parasites with possible deletions in *pfhrp2* or *pfhrp3* genes. If the detected deletions are true, it is difficult to infer whether they existed at the time of original infection, or resulted from a subsequent laboratory adaptation. Illumina short read sequencing used to generate the genomic data also poses a limitation of this study because of its propensity to generate uneven coverage profiles across the genome (e.g., high coverage at certain loci and little to no coverage at other loci) thus possibly influencing the probability of correctly detecting possible gene deletions. The origin of such coverage heterogeneity can be due simply to library preparation and sequencing bias of smaller fragments. The highly AT-enriched *P*. *falciparum* genome also is known to generate biases in the coverage distribution^23^. In theory, the use of alternative sequencing platforms that are able to generate longer reads (i.e., pacbio or minION) could produce higher quality coverage data either by mapping onto the 3D7 reference genome or by *de novo* assembly of the sampled genomes. In practice, this study used publicly available genomic data generated elsewhere and, therefore, it was not possible to perform alternative genomic approaches. However, the exclusion of genomic samples with sufficiently large proportions of regions with zero coverage minimized the chance of inferring deletions in the *pfhrp2* and *pfhrp3* loci when there are in fact none. A limitation of our analysis is the possibility of underestimating the true number of deletions due to challenges in mapping these genes. *Pfhrp2* and *pfhrp3* genes share high sequence homology; therefore mapping algorithms such as the bwa-mem, used in the pre-processing of the samples, usually allocate reads randomly to different homologous sequences present in the genome. If this is the case, however, the existence of a deleted *pfhrp2* gene in presence of fully functional *pfhrp3*, although limiting the power of genomic analysis, is unlikely to have strong consequences in the RDT testing as shown by our model (Fig. 3).

These data also confirm the role of parasitaemia in generating RDT signal by the *pfhrp2*−/*pfhrp3*+ parasites. The model shows that parasites lacking *pfhrp2* produce positive HRP test bands at similar parasitaemia to those with the intact gene particularly at higher parasitaemia, supporting a previous report of the absence of compensatory increase of the HRP3 protein^21^. This was evident on the six *pfhrp2*-negative samples, which were also negative by RDT and microscopy but positive for *P*. *falciparum* by qPCR at very low parasitaemia. We did not identify these parasites as lacking *pfhrp2* since the negative result was more likely due to low parasitaemia, and a criterion for establishing presence of deletion is the confirmation of parasites by microscopy^16^. We identified the only sample with *pfhrp3*-negative result as deletion, as the sample had adequate parasitaemia by qPCR, and microscopy might have been false negative as the slide was only read once. Further study of samples with adequate levels of parasitaemia will help to confirm these findings.

The sensitivity and clinical performance of HRP2-based RDTs in Kenya are not expected to be affected by the findings of this study. In this study, HRP2-based RDT results were positive in the presence of *P*. *falciparum* infection regardless of *pfhrp2* gene status, particularly at higher parasitaemia (above approximately 1360 p/µL), presumably due to antigen-antibody cross-reactivity with HRP3 or other proteins. In addition, parasitaemia tends to be higher in symptomatic patients and therefore the effect of *pfhrp2* deletion on patient diagnosis is expected to be even lower. Reassuringly, a recent study that evaluated eight different HRP2-based RDTs among 500 symptomatic and asymptomatic individuals in western Kenya, and reported sensitivity compared with microscopy of 90–95%^7^.

These data illustrate the difficulty of amplifying *pfhrp2* and *pfhrp3* genes in samples with submicroscopic parasite density, and support the guidance to rule out low parasitaemia as the cause of negative PCR results before reporting *pfhrp2* and *pfhrp3* deletions^16^. These results also highlight that to determine the true prevalence of *pfhrp2* deletions in the population, the surveys should include HRP2 RDT-positive, as well as HRP2-RDT negative samples. Furthermore, investigations should include PCR testing for both *pfhrp2 and pfhrp3*, because this may be the most accurate way to estimate the risk of false negative RDT samples due to *pfhrp2* deletions in clinical settings. A limitation of this study is lack of HRP ELISA data to quantify HRP antigenemia and further PCR findings. The difference between *pfhrp2*+/*pfhrp3*+ and *pfhrp2*−/*pfhrp3*+ samples particularly at lower parasitaemia could be due to difference in the amount of antigens generated and in relative affinity of HRP2 and HRP3 for the RDT antibodies, which we could not measure and which our model (Fig. 3) did not take into account. This study is also limited in that the genomic and genotype data are not from the same samples or from the same area in Kenya, although malaria endemicity in both regions is similar^24^.

In summary, results of this study demonstrate that *P*. *falciparum* isolates lacking *pfhrp2* genes are present in Kenya, they infect humans, and cause symptomatic disease but they are also still detectable by at least some brands of HRP2-based RDTs in the presence of an intact *pfhrp3* expressing antigen levels found at parasitaemia ≥1000p/µL However, these findings warrant further systematic study to understand factors driving *pfhrp2* deletions; to establish the true prevalence and distribution of *pfhrp2* and *pfhrp3* deletions and to monitor for false negative HRP2 based RDTs and the potential implications for diagnostic testing strategies.

## Materials and Methods

### Study populations

A total of 274 blood samples were collected between January and July 2014 from asymptomatic children aged 5 to 12 years attending schools near Mbita, western Kenya, as part of a study on mosquito behavior and the odor profile of malaria infected individuals. The study received approval from the Kenya Medical Research Institute Ethical Review Committee (KEMRI/RES/7/3/1) and the London School of Hygiene and Tropical Medicine Ethics Committee (reference 8510). Written informed consent was obtained from a parent or guardian of each participating child. All experiments were performed in accordance with relevant guidelines and regulations.

Data from 61 *P*. *falciparum* samples from Kilifi District, eastern Kenya, were obtained from the MalariaGEN *Plasmodium falciparum* Community Project as described in Genomic epidemiology of artemisinin resistant malaria^25^. These genomic data were generated from parasite samples which were obtained from pediatric patients enrolled in a clinical trial as described elsewhere, and then cultured in the lab until full adaptation^26^.

### Blood sample preparation of the Mbita samples

Finger-prick blood from each participant was analyzed by light microscopy for the presence of parasites; applied to an RDT (SD Bioline Malaria Ag P.f/Pan test, HRP2/pan-pLDH, catalog number 05FK60, Standard Diagnostics, Hong Dong, Korea); and used to prepare dried blood spots (DBS) which were stored at room temperature until they were shipped to London School of Hygiene & Tropical Medicine (LSHTM). Slides were read by a study team microscopist trained at the Walter Reed Project in Kisumu, Kenya, and a subset (10%) of slides were re-read independently by a second microscopist at the LSHTM Malaria Reference Laboratory, London, UK. For the double-read subset, correlation between the two readers was moderate (r = 0.76, *P* < 0.001). RDTs were prepared and interpreted by a study team member according to the manufacturer’s instructions.

The RDTs and DBS were sent to LSHTM for molecular analysis. RDTs were available for all 274 samples, while DBS were available for 141. (Investigators for the primary entomology study modified their protocol to include DBS only midway through the study). Both template types were available for 141 samples, which were used as a comparison to validate the extraction of DNA from RDTs and subsequent quantification of parasitaemia by qPCR, with no significant difference observed (A Robinson, in preparation).

DNA was extracted using a robotic extraction system (QIAsymphony) using DSP DNA mini kit according to the manufacturer’s protocol (QIAGEN, Germany). In order to reduce costs, we validated the DSP DNA mini kit instead of using the manufacturer-recommended investigator kit for DNA extraction from DBS and RDTs with no significant difference (A Robinson, in preparation). DBS were pre-treated according to the manufacturer’s manual before DNA extraction. Briefly, 1 × 3 mm diameter punch from the DBS or three cuts of the nitrocellulose strip of the RDTs^27^ were placed in the deep-well plate. Buffer ATL (180 µL) and proteinase K (20 µL) were added to each well and mixed by thermomixer at 900 rpm at 56 °C for 15 min. The deep-well plate was then placed directly into the sample compartment of the QIAsymphony for DNA extraction. The extracted DNA samples were stored at −20 °C for further molecular biology investigation.

### Amplification of *pfhrp2* and *pfhrp3*

The presence of parasites was detected by standard PCR targeting the 18S ribosomal RNA gene of *P*. *falciparum* (*18SrDNA*), which has a limit of detection of 0.1 parasite per µL (0.000002% parasitaemia)^28,29^. All samples positive by *18SrDNA* assay were then subjected to *pfhrp2 and pfhrp3* genotyping using previously published PCR conditions and primers as described by Baker *et al*.^2,8^. The elongation temperature was modified to 60 °C and annealing temperature to 50 °C in the repeated experiments of *pfhrp2* and *pfhrp3* (Fig. 2)^11^.

### Confirmation of *pfhrp2* and *pfhrp3* deletion

To verify the presence of parasites in the *pfhrp2*-negative samples, PCR assays targeting *msp1* and *msp2* genes were amplified using previously reported methods^30^. Microscopy-positive samples with positive results for *18SrDNA*, *msp1* and *msp2* assays but negative results for *pfhrp2* /*pfhrp3* were considered *pfhrp2* /*pfhrp3*-deleted after excluding low parasitaemia in order to avoid incorrect deletion calls (See Fig. 2 for algorithm).

### Parasitaemia estimation by qPCR

To explore the role of low parasitaemia in apparently negative *pfhrp2* /*pfhrp3* genotyping results, the parasitaemia of each sample was quantified using a previously published *PgMET* qPCR assay, which has a limit of detection of 5 parasites per µL^31,32^. All samples, including samples that were negative by *18SrDNA* assay, were included in order to capture any false negatives. International Standard was used as a positive control (calibrator) with the original sample (9.6% parasitaemia) diluted to 0.0096% (~500 parasite per µL)^33^. The accuracy of this quantification was then compared to parasitaemia generated by microscopic examination of blood films, when available.

### Statistical analysis of field samples

The main outcomes for this study were results of HRP-based RDT, results of *pfhrp2* and *pfhrp3* PCR, and parasitaemia as determined by microscopy and qPCR. Parasite density measured by qPCR for each sample was calculated using International Standard (IS) as a calibrator^32,33^, which was used as factor to calculate the parasitaemia of each sample. Parasitaemia was compared between groups (e.g. RDT-positive vs RDT-negative samples, *pfhrp2*-positive vs *pfhrp2*-negative) using Kruskal-Wallis rank test. Samples below the qPCR limit of detection (≤5 parasites per µL [p/μL]) were excluded from further *pfhrp2* and *pfhrp3* deletion call. Generalized linear models for binomial responses were used to study the probability of RDT positive with log parasitaemia, *pfhrp2*/*pfhrp3* status, as described in S1 Table. The best model was selected according to the Akaike’s and Bayesian Information Criteria. The statistical validation of this model was made via the Hosmer-Lemeshow goodness-of-fit test using 5 bins to calculate the quantiles. The area under the curve (AUC) of the receiver operating characteristic curve predicted by the model was also estimated in order to assess the predictive power of the model. For the Hosmer-Lemeshow test, a p-value > 0.05 indicated a good fit of the model while an estimate of the AUC close to 1 provided evidence for a very good prediction of the observed RDT positivity data by the model.

Data analysis was performed using either STATA (version 14, USA), or R (version 3.3.2, http//www.r-project.org). In the R software, the packages ResourceSelection and pROC were specifically used to perform the Hosmer-Lemeshow test and to estimate AUC, respectively.

### Analysis of *pfhrp2* and *pfhrp3 genes* using whole-genome sequencing data

The 61* P*. *falciparum* samples from Kilifi underwent whole-genome sequencing and were processed as described in detail elsewhere^34^. Briefly, for each sample, the raw sequence reads were aligned onto the 3D7 reference genome (version 3.0) using the bwa-mem short read alignment algorithm. For each sample, the total number of mapped reads per position (coverage) was calculated for the whole genome. The genomic analysis of *pfhrp2* and *pfhrp3* genes (PF3D7_0831800 and PF3D7_1372200) was restricted to position 1,372,236-1,377,299 of chromosome 8 (*pfhrp2* locus), and to position 2,838,727-2,843,703 of chromosome 13 (*pfhrp3* locus). These two genomic regions included the coding regions of each gene and the respective 2 kb flanking regions (to either side).The corresponding coverage data was then analyzed to identify samples with possible deletions of these genes, using a statistical description of the coverage data. The application of existing genomic tests for detecting deletions using coverage data were outside the scope of this paper as they typically require integration of information from the entire genome^23,35^.

### Data availability

The datasets generated during and/or analysed during the current study are available from the corresponding author on reasonable request.

## Electronic supplementary material

Supplementary Table S1. Data generated using RDT, pfhrp2, pfhrp3 and parasitaemia (>5 parasites per microliter, n=91).
