# Supplementary material for: Plasmodium falciparum parasites with histidine-rich protein 2 (pfhrp2) and pfhrp3 gene deletions in two endemic regions of Kenya
Source: Sci Rep. 2017 Nov 7;7:14718. doi: 10.1038/s41598-017-15031-2 (PMC5677122; doi:10.1038/s41598-017-15031-2)
Supplement: Supplementary file 1 — Supplementary Table S1. Data generated using RDT, pfhrp2, pfhrp3 and parasitaemia (>5 parasites per microliter, n=91). [file 41598_2017_15031_MOESM1_ESM.pdf]

Plasmodium falciparum parasites with histidine-rich protein 2 (pfhrp2) and pfhrp3 gene deletions in two endemic regions of Kenya

Khalid B Beshir*[1], Nuno Sepúlveda[1,2], Jameel Bharmal[1], Ailie Robinson[1], Julian Mwanguzi[1], Annette Obukosia Busula[3], Jetske Gudrun de Boer[4], Colin Sutherland[1], Jane Cunningham[5] and Heidi Hopkins[1]

1. London School of Hygiene and Tropical Medicine, London, UK
2. Centre for Statistics and Applications of University of Lisbon, Lisbon, Portugal
3. International Centre of Insect Physiology and Ecology, Nairobi, Kenya. Current affiliation: Kaimosi Friends University College, Kaimosi, Kenya.
4. At the time of the work reported here: Laboratory of Entomology, Wageningen University, Droevendaalsesteeg 1, 6708 PB Wageningen, The Netherlands. Current affiliation: Netherlands Institute of Ecology, Droevendaalsesteeg 10, 6708 PB Wageningen, The Netherlands
5. Global Malaria Programme, World Health Organization (WHO-GMP), Geneva, Switzerland

*Correspondence to Khalid.Beshir@lshtm.ac.uk

Key words: hrp2 deletion, *pfhrp2*, *pfhrp3*, malaria, *Plasmodium falciparum*, rapid diagnostic test, RDT, HRP2, HRP3, histidine rich protein

Supplementary material

Supplementary Table S1. Data generated using RDT, *pfhrp2*, *pfhrp3* and parasitaemia (>5 parasites per microliter, n=91).

Model	Model comparison		Parameter estimates (Standard Error)			
	AIC	BIC	Intercept (a)	hrp2 (b)	hrp3 (c)	Parasitaemia (d)
Logit	34.22	44.22	-2.45 (1.47)	3.33 (1.21)	-0.03 (1.36)	0.72 (0.34)
Probit	34.36	44.36	-1.55 (0.83)	1.65 (0.55)	0.45 (0.75)	0.36 (0.17)
Complementary log-log	32.74	42.74	-2.50 (1.08)	1.57 (0.55)	1.09 (0.73)	0.35 (0.16)

Analysis of the probability of RDT positivity as function of parasitaemia (in log scale), presence of *Plasmodium falciparum* histidine rich 2 and 3 (*pfhrp2* and *pfhrp3*) respectively) genes using different generalized linear models for binomial responses (logit, probit and complementary log-log). The general formulation of these models was the following:

$$g(x) = a + b \times \text{hrp2} + c \times \text{hrp3} + d \times \log(Parasitaemia),$$

where hrp2 and hrp3 are binary covariates indicating the presence of *pfhrp2* and *pfhrp3*, respectively, $g(x) = log\, p/(1-p)$ (logit), $g(x) = \Phi(p)$ (probit), and $g(x) = \log(-\log(1-p))$(complementary log-log). The coefficients *a*, *b*, *c* and *d* were estimated by maximum likelihood method using the *glm* function for R. Models were compared with each other via Akaike's information criterion (AIC) and Bayesian Information Criterion (BIC), where the best model for the data is the one with the lowest estimate for each measure.
